# Burden, etiology and predictors of visual impairment among children attending Mulago National Referral Hospital eye clinic, Uganda

**DOI:** 10.4314/ahs.v17i3.31

**Published:** 2017-09

**Authors:** Patience Kinengyere, Samuel Kizito, John Baptist Kiggundu, Anne Ampaire, Geoffrey Wabulembo

**Affiliations:** Makerere University College of Health Sciences

**Keywords:** Visual impairment, Mulago National Referral Hospital, Eye clinic, Uganda

## Abstract

**Background:**

Childhood visual impairment (CVI) has not been given due attention. Knowledge of CVI is important in planning preventive measures. The aim of this study was determine the prevalence, etiology and the factors associated with childhood visual impairment among the children attending the eye clinic in Mulago National Referral Hospital.

**Methods:**

This was a cross sectional hospital based study among 318 children attending the Mulago Hospital eye clinic between January 2015 to March 2015. Ocular and general history was taken and patient examination done. The data generated was entered by Epidata and analyzed by STATA 12.

**Results:**

The prevalence of CVI was 42.14%, 134 patients with 49 patients (15.41%) having moderate visual impairment, 45 patients (14.15%) having severe visual impairment and 40 patients (12.58%) presenting with blindness. Significant predictors included; increasing age, delayed developmental milestones and having abnormal corneal, refractive and fundus findings.

**Conclusion:**

There is a high burden of visual impairment among children in Uganda. It is vital to screen all the children presenting to hospital for visual impairment. Majority of the causes of the visual impairment are preventable.

## Introduction

Visual impairment includes low vision as well as blindness. Low vision is defined as visual acuity of less than 6/18, but equal or better than 3/60, or a corresponding visual field loss to less than 20 degrees in the better eye with best possible correction. Blindness is defined as visual acuity of less than 3/60, or corresponding visual field loss to less than 10 degrees in the better eye with the best possible correction^1^

Globally, 1.4 million children are estimated to be blind, one fifth of whom are from Africa.^2^ A child goes blind every minute and the most at-risk are those children below 5 years of age. About 60% of the children die within one year of becoming blind.^3^ Childhood visual impairment has a lifelong impact to the patient, which makes it a significant problem.^4^ In the Low and Middle Income Countries (LMIC), up to 72% of the blindness is preventable while up to 31% is treatable.^2^

World Health Organization WHO through “The Right to Sight” global initiative prioritizes childhood blindness as one of the five conditions for control by 2020.^5^ Approximately 90% of visually impaired children in LMIC do not attain formal education. Childhood blindness has devastating implications not only for the affected child but the family as well. The devastation is lifelong and profoundly impacts negatively on educational, employment, personal, and social prospects.^6^. The quality of life of the visually impaired children is tremendously compromised given the number of Blind Person Years (BPYs) ahead of them.^7^

Despite the devastation childhood visual impairment CVI can cause and the noticeable increase in the number of children presenting with CVI in Mulago National Referral Hospital over recent years, the condition has not been given due attention. There is paucity of literature regarding the burden of visual impairment in Uganda. Our study aimed at assessing the burden, etiology and predictors of visual impairment among children presenting to Mulago hospital eye clinic. The findings from our study have given some insight into the magnitude of the burden of childhood visual impairment in Uganda and highlight key areas in reducing preventable causes of blindness in children.

## Methods

### Study design and study setting

This was a cross sectional study conducted in the eye clinic of Mulago National Referral Hospital.

Mulago National Referral Hospital is located in Kampala, the capital city. It has a capacity of about 1500 beds. The hospital also serves as a teaching hospital for Makerere University College of Health Sciences. The hospital has two eye clinics. The ophthalmology department has a clinic run by ophthalmic clinical officers. It is the first point of contact with the patients unless they are referrals from the other health units. The second clinic is a consultation clinic and is run by the ophthalmologists. The department has ten dedicated ophthalmologists, two of whom are pediatric ophthalmologists.

### Study population

We conducted this study among patients below the age of 18 years who presented to the eye clinic at Mulago National Referral Hospital during the months of January to March 2015. We excluded any patient who was too sick to withstand the rigorous examinations during the study period.

### Sampling and sample size calculation

We employed systematic sampling, taking every second pediatric patient seen at the clinic each day. The clinic receives an average of 75 pediatric patients per week. The first patient was randomly selected each day, and then every second pediatric patient eligible for the study was enrolled upon consenting. The research assistant helped to identify the proposed patients.

Using the the Kish Leslie's formula^8^, we estimated the sample size of 318 patients for the study using a prevalence of 29.3 % .^9^

### Study procedures

We conducted a baseline visual acuity to ascertain whether the child had visual impairment. The visual acuity test used was according to the age of the child.

Children 5 years and above who could read were assessed using the Snellen's chart. Pre-verbal children were tested using ‘preferential looking’ techniques. Children 18 months to 60 months were assessed using the Cardiff test. In the children 6–24 months, Lea Gratings were used to test visual acuity.^10^

All the study participants had a detailed history taken, general physical examination and ocular examination done. Standard ophthalmic equipment was used to do the examinations. These included tape measure to measure the head circumference, ophthalmoscope, retinoscope, slit lamp, examining torch, lid speculum and prism bars. Data was collected using a questionnaire. Examination of the lids, conjunctiva, cornea, anterior chamber, pupil and iris was done using a torch and slit lamp. Dilating of the pupils was done with cyclopentolate or tropicamide eye drops. Dilated indirect ophthalmoscopy was done in all study participants. Clycloplegic retinoscopy was done on all the study participants.

Any ocular anomaly detected during the patient assessment was documented and managed as appropriate. Any non-ocular anomaly detected during the patient assessment was documented and the needed specialty consulted on the course of management.

### Study variables

#### Dependable variable: Childhood visual impairment.

Visual impairment included low vision as well as blindness. We defined low vision as visual acuity of less than 6/18, but equal or better than 3/60, or a corresponding visual field loss to less than 20 degrees in the better eye with best possible correction. We defined blindness as visual acuity of less than 3/60, or corresponding visual field loss to less than 10 degrees in the better eye with the best possible correction.^1^ We defined visual impairment to include low vision and blindness.

**Independent variables:** Socio-demographic factors: age, sex, address, socio-economic factors: occupation of parents, level of education of the parents, education of the child and clinical factors like history of systemic illnesses and ocular illnesses.

#### Data management and analysis.

Data collected was double entered in to the computer software using EpiData version 3.1. Data was cleaned and exported for analysis. We analyzed the data using the STATA version 12. We summarized continuous data using measures of central tendency. Categorical variables were summarized into frequencies and percentages. Comparisons between the continuous variables were done using student t-tests or the Mann-Whitney U test depending on the distribution of the data. While for the categorical data, we employed the Chi-squared tests.

To assess for the associated factors, we applied logistic regression mathematical modelling techniques. All the factors with a p value ≤ 0.20 at bivariate analysis were included in the multivariate logistic regression as well.

**Ethical considerations.** We obtained approval to conduct the study from the Makerere University School of Medicine Research and Ethics Committee and the Uganda National Council for Science and Technology. We obtained Informed signed consent from the care takers of the children and additional assent from the children who were 8 years and above.

## Results

Of the 318 patients enrolled in our study, 170 (53.46%) were females. Majority of the participants, 129 (40.57%) were below 5 years of age. Of all the parents to the participants, 93.40% of the parents/ guardians had formal education and 90.57% had a source of employment. Details are shown in table below.

### Antenatal, perinatal and post-natal history.

Majority of the mothers to the participants, 296 (93.1%) attended antenatal care at least once during the pregnancy and 76 (23.9%) had history of febrile illness during pregnancy.

Of all the deliveries, 295 (92.8%) were conducted by a trained personnel. A large proportion, 306 (96.2%) of the participants had normal weight for age on presentation and 277 (87.1%) of the participants had normal head position. Details are highlighted in table below.

### Diagnosis presented by the study participants

From table 3, the commonest diagnosis made for the participants was ocular trauma, 61 19.2%. Anatomically, the commonest ocular abnormality was pupillary reaction disorders found among 76 23.9% of all the children.

**Table 3:** Table showing classification of the diagnoses by etiology among 318 children below 18 years attending Mulago Hospital eye clinic, 2015.

Variable	Total n (%)	Normal vision	Visual impairment	p value
Etiological classification of Refractive errors	22 (6.9)	14 (4.4)	8 (2.5)	0.0
Infections	19 (5.9)	8 (2.5)	11 (3.5)	0.0
Trauma	61 (19.2)	15 (4.7)	46 (14.5)	0.00
Inflammation	46 (14.2)	45 (14.2)	1 (0.8)	0.00
Neoplasm	10 (3.1)	6 (1.9)	4 (1.5)	0.00
Benign legions	29 (9.1)	29 (9.1)	0 (0.0)	0.00
Cataract	17 (5.3)	5 (1.6)	12 (3.8)	0.00
Squint	41 (12.9)	30 (9.4)	11 (3.5)	0.00
Congenital ocular anomalies	6 (1.9)	0 (0.0)	6 (1.9)	0.00
Others*	67 (21.1)	31 (16.9)	36 (26.9)	0.00
Anatomical classification of Abnormal External eye	50 (15.7)	42 (13.2)	8 (2.5)	0.00
Abnormal corneal exam	57 (17.9)	6 (1.9)	51 (16.0)	0.00
Abnormal pupillary reaction	76 (23.9)	1 (0.3)	75 (23.6)	0.00
Abnormal fundus findings	30 (9.4)	3 (0.9)	27 (8.5)	0.00

* other etiologies include: tumor, plosis, chalazion, demoid cyst, corneal ulcer, optic neuropathy, lid hemangioma, foreign body, molluscum contagiosum, nasolacrimal duct obstruction, keratoconus, nystagmus, cortical blindness, entropion, sickle cell disease, conjunctival growth, buphalmos, microphthalmos, microcornea, anophthalmos, diabetic retinaopathy, retinitis pigmentosa, uveitis, symblepharon

### Visual impairment

In total, we found 134 (42.1%) of the participants having visual impairment, 49 (15.4%) had moderate visual impairment, 45 (14.2%) had severe visual impairment and 40 (12.6%) had blindness. Only 184 (57.9%) participants had normal vision on presentation. We found high prevalence of visual impairment among children with delayed developmental milestones, among those with history of convulsions, and those with low birth weight. Details are in table 4.

**Table 4:** Prevalence of visual impairment among 318 children below 18 years attending Mulago Hospital eye clinic, 2015.

Variable	Number	Prevalence 95% CI
**Overall visual impairment**	134	42.8 36.8 – 47.7
Age		
Below 5 years	129	32.6 24.9 – 41.2
5 to 12 years	120	45.0 36.2 – 54.1
Above 12 years	69	55.1 43.0 – 66.6
**Duration of the presenting complaint**		
Less than a week	85	50.0 24.7 – 97.5
Lasted a week or more	233	50.0 24.7 – 97.5
**Use of local medications for eyes**		
Used local eye medicine	26	53.8 33.8 – 72.7
Did not use local eye medicine	292	41.1 35.6 – 46.7
**Antenatal attendance**		
Mother attended antenatal care	296	37.5 8.7 – 79.2
Did not attend antenatal care	22	41.9 36.4 – 47.6
**Fever during pregnancy**		
Had a fever during pregnancy	76	42.5 36.2 – 49.1
No fever during pregnancy	242	39.5 28.9 – 51.1
**Birthplace**		
Had a birth attendant	295	42.136.6 – 47.7
Delivered in absence of a birth attendant	17	33.3 0.7 – 99.7
**Attaining developmental milestones**		
Attained milestones on time	291	39.2 33.7 – 44.9
Delayed milestones	27	74.1 53.2 – 87.8
**History of convulsion**		
Patient has no history of convulsions	307	39.9 34.4 – 45.5
Had history of convulsions	11	81.8 42.0 – 96.5
**Birth weight**		
Underweight	6	85.7 25.7 – 99.0
Normal weight	306	40.7 35.5 – 46.3
**Gestation age**		
Term delivery	304	40.8 35.3 – 46.4

### Predictors of visual impairment

As shown in table 5, we found the following factors to be significantly associated with visual impairment. Age, delayed attainment of the developmental milestones, having abnormal pupillary reaction, corneal exam as well as having abnormal fundus examination findings.

**Table 5:** Factors associated with visual impairment among 318 children below 18 years attending Mulago Hospital eye clinic, 2015.

	Bivariate Analysis		Multivariate Analysis	
**Characteristic**	**eOR (95% CI)**	**P value**	**aOR (95% CI)**	**P value**
Age				
Below 5 years	1		1	
5 to 12 years	1.7 (1.0 – 2.8)	0.05	5.5 (1.4 – 22.4)	**0.02**
Above 12 years	2.5 (1.4 – 4.6)	0.00	14.6 (3.2 – 66.2)	**0.00**
Patient is a male	1.2 (0.8 – 1.9)	0.41	1.5 (0.6 – 3.5)	0.37
Antenatal history				
PC more than 7 days	0.6 (0.3 – 1.1)	0.09		
Did not use traditional eye medicine	0.6 (0.3 – 1.3)	0.21		
Did not attend antenatal care	0.8 (0.2 – 3.6)	0.80		
No Fever during pregnancy	1.1 (0.7 – 1.9)	0.64		
Mother had no systemic illness	1.8 (0.4 – 9.5)	0.48		
Mother was not taking any medications	1.8 (0.4 – 9.5)	0.48		
Perinatal history				
No trained personnel at delivery	1.2 (0.4 – 3.6)	0.77		
Baby born preterm	1.9 (0.4 – 8.8)	0.39		
Birth weight				
Normal	1			
Underweight	2.9 (0.5 – 16.2)	0.22		
Given Oxygen therapy after birth	1.8 (0.8 – 4.1)	0.19	1.2 (0.2 – 6.4)	0.81
Patient has history of convulsion	6.8 (1.4 – 31.9)	0.02		
Developmental milestones				
Delay developmental milestone	4.4 (1.8 – 10.8)	0.00	19.1(1.0 – 404.9)	**0.05**
Abnormal Weigh for age	7.3 (1.6 – 34.1)	0.01	0.8 (0.0 – 34.1)	0.88
Abnormal Height for age	6.6 (1.4 – 30.8)	0.02		
Head big for age	0.5 (0.1 – 4.6)	0.52		
Head Position				
Tilted	1			
Normal	0.3 (0.1 – 0.6)	0.00		
Chin left	2.3 (0 .4– 13.0)	0.36		
Ocular examination findings normal	1			
Has abnormal cyc exam	0.2 (0.1 – 0.5)	0.00		
Abnormal corneal exam	16.4 (6.6 – 40.0)	0.00	7.0 (1.7 – 29.5)	**0.00**
Abnormal refraction	9.9 (5.0 – 19.7)	0.00	9.5 (3.9 – 23.1)	**0.00**
Has abnormal pupillary reaction	130.3 (17.6 – 967.2)	0.00		
Abnormal fundus exam	15.2 (4.5 – 51.4)	0.01	9.3 (1.9 – 45.1)	**0.01**

## Discussion

### Prevalence of childhood visual impairment.

We found a high burden of visual impairment among the children. For every 10 patients seen in the pediatric eye clinic, 4 will present with visual impairment. This is a high prevalence compared to a similar setting. In Nigeria, they reported a prevalence of 29.3%^9^. However unlike our study, this was a community based study. Only those in need of the hospital services come to hospital compared to the community where everyone is seen regardless of their need for the given service.

Children between 5 to 12 years were 1.7 times more likely to suffer from visual impairment and children above 12 years were 2.5 times more likely to suffer from visual impairment as compared to the children below 5 years. Children who had sustained ocular trauma were 5.3 times more likely to suffer from visual impairment. Other factors with a strong association to visual impairment included; refractive errors, cataract, ocular infections and tumors. All these can be preventable or managed to prevent visual impairment in a child.

### Etiology for childhood visual impairment.

Trauma was among the top etiologies for childhood visual impairment during the study period. This correlates with the findings in the study on causes of childhood visual impairment in East Africa^11^. The study showed a high proportion of visually impaired children with corneal pathology, with most cases being due to trauma. Ocular trauma is the cause of blindness in approximately half a million people worldwide. Trauma is often the most important cause of unilateral loss of vision, particularly in developing countries.^12^

Refractive errors were found to be important with regards to visual impairment with 8 of the 22 children that presented with refractive errors being visually impaired with an overall occurrence of 2.52%.

### Childhood visual impairment by anatomical site

Among the children that were reviewed, extra ocular pathology, corneal pathology, pupillary defects and fundus pathology were found to be significant for visual impairment. This correlates with the WHO report on childhood visual impairment in the context of the VISION 2020 where corneal and retinal pathologies were found to be significant association with childhood visual impairment. Causes of severe childhood visual impairment and blindness were analyzed across the global social economic spectrum and retinal pathology (29%), cornea pathology (15%), whole globe (16%), lens (12%), optic nerve 12% and were found to be significant^13^.

## Conclusion

Our study has shown a high burden of visual impairment among children in Uganda. These findings indicate that it is vital to screen all the children presenting to hospital for visual impairment. Majority of the causes of the visual impairment are preventable. We however recommend a wider population based study on the prevalence and causes of childhood visual impairment to give a more detailed insight in order for policies to be amended to emphasize childhood visual impairment.

## Limitations

Our study was hospital based in a National Referral Hospital eye clinic which is a specialized clinic. This reduces the generalizability of our findings and does not give a general picture of the prevalence of childhood visual impairment of the general population.

## Figures and Tables

**Table 1:** Socio-demographic characteristics of 318 children below 18 years attending Mulago Hospital eye clinic, 2015

Variable	Total n %	Normal vision	Visual impairment	P value
Age years				
Below 5 years	129 40.3	87 27.4	42 13.2	**0.01**
5 to 12 years	120 37.7	66 20.8	54 16.9	
Above 12 years	69 21.7	31 9.8	38 11.9	
Patient is female	170 (53.5)	102 (32.1)	68 (21.4)	0.41
Ethnicity				
Bantu	270 (84.9)	156 (40.1)	114 (35.9)	0.74
Hamites	13 (4.1)	6 (1.9)	7 (2.2)	
Nilo-hamites	12 (3.8)	7 (2.2)	5 (1.6)	
Nilotic	23 (7.2)	15 (4.7)	8 (2.5)	
Child stays with parent/guardian	304 (95.6)	175 (55.3)	129 (40.6)	0.96
Parent has formal education	297 (93.6)	178 (55.9)	119 (37.4)	**0.01**
Parent's guardian's occupation				
Unemployed	30 (9.4)	13 (4.1)	17 (5.4)	0.18
Employed	288 (90.6)	171 (53.8)	117 (36.8)	
Child's education				
Preschool age	118 (37.1)	78 (24.5)	40 (12.6)	**0.00**
In school	187 (58.8)	105 (33.0)	82 (25.8)	
Not in school	13 (4.1)	1 (0.3)	12 (3.8)	
Used traditional eye medicine	26 (8.8)	12 (3.8)	14 (4.4)	0.21

**Table 2:** Antenatal, natal and postnatal history of 318 children below 18 years attending Mulago Hospital eye clinic, 2015.

Variable	Total D (%)	Normal vision	Visual impairment	P value
Attended antenanal care	296 (93.1)	172 (54.1)	124 (39.0)	0.80
Had febrile illness during pregnancy	76 (23.9)	46 (14.5)	30 (9.4)	0.64
Mother has systemic illness	7 (2.2)	5 (1.6)	2 (0.6)	0.47
Assistant at delivery				
Trained	295 (92.8)	170 (53.5)	125 (39.3)	0.91
Not trained	17 (5.4)	12 (3.8)	5 (1.6)	
Unknown	6 (1.9)	2 (0.6)	4 (1.3)	
Baby was full term at delivery	304 (95.6)	179 (56.3)	125 (39.3)	0.39
Birth weight				
Normal	305 (95.6)	181 (56.3)	124 (39.0)	**0.03**
Underweight	6 (1.9)	2 (0.6)	4 (1.3)	
Unknown	7 (2.2)	1 (0.3)	5 (1.6)	
Oxygen therapy after delivery				
No oxygen given	288 (90.6)	172 (54.3)	116 (36.5)	0.18
Oxygen was given	24 (7.6)	11 (3.5)	13 (4.1)	
Has history of convulsions	11 (3.5)	2 (0.6)	9 (2.8)	**0.01**
Delayed developmental milestones	27 (8.5)	7 (2.2)	20 (6.3)	**0.01**
Anthropometry				
Normal weight for age	306 (96.2)	182 (57.2)	124 (39.0)	**0.03**
Head size				
Normal	308 (96.9)	181 (56.9)	127 (39.9)	**0.01**
Big for age	10 (3.1)	3 (0.9)	7(2.2)	
Abnormal Head position	41 (12.9)	13 (4.1)	28 (8.8)	**0.00**

## Abbreviations

ANC: Antenatal care
CVI: Childhood Visual Impairment
MDGs: Millennium Development Goals
PBYs: Person Blind Years
ROP: Retinopathy of Prematurity
SVI: Severe Visual Impairment
TEM: Traditional eye medicine
WHO: World Health Organization
